# Favourable outcome of multisystem venous thrombosis associated with novel *SERPINC1* mutation after treated with dabigatran: a case report with 7-year follow-up

**DOI:** 10.1186/s12959-022-00446-3

**Published:** 2022-12-28

**Authors:** Teng Huang, Yu Liu, Xiaofeng Jiang, Wei Zhang, Honglian Zhou, Qi Hu

**Affiliations:** 1 Department of Geriatrics, Tongji Hospital, Tongji Medical College, Huazhong University of Science and Technology, 430030 Wuhan, PR China; 2 Department of General medicine, Tongji Hospital, Tongji Medical College, Huazhong University of Science and Technology, 430030 Wuhan, PR China; 3 Department of General medicine, Yangxin County General Hospital, 435200 Yangxin, PR China; 4 Department of Hematology, Tongji Hospital, Tongji Medical College, Huazhong University of Science and Technology, 430030 Wuhan, PR China

**Keywords:** Thrombophilia, Venous thrombosis, Antithrombin deficiency, *SERPINC1*, Dabigatran

## Abstract

**Background:**

Mutations in *SERPINC1* lead to deficiency in antithrombin (AT) which is an endogenous anticoagulant of normal hemostasis and could result in venous thromboembolism (VTE).

**Case presentation:**

A 61-year-old male patient with recurrent thrombosis returned to the hospital with multiple cerebral thrombosis after voluntary cessation of dabigatran therapy. Laboratory tests revealed a type I AT deficiency in this patient and further whole exome sequencing (WES) identified a novel heterozygous frameshift duplication (c.233_236dup, p.Val80Alafs*26) in *SERPINC1* gene. Long-term dabigatran treatment was given and no recurrence or side effects were found within the followed 5 years.

**Conclusion:**

A multisystem VTE patient with a novel *SERPINC1* mutation (c.233_236dup, p.Val80Alafs*26) reached a favourable outcome after dabigatran treatment.

## Background

Thrombophilia caused by inherited or acquired conditions is a group of diseases predisposing patients to thrombosis [1]. The main clinical symptom is recurrent venous thromboembolism (VTE). VTE typically occurs in deep veins of the legs and arms, but sometimes the thrombosis occurs in uncommon sites, such as the splanchnic veins, cerebral veins and retinal vein [2]. Thrombophilia is etiologically multifactorial and involves an interaction between inherited and acquired factors. The acquired risk factors include antiphospholipid antibody syndrome, malignancy, oral contraceptive use, hormonal replacement therapy, surgery, obesity, smoking, prolonged travel, immobility and pregnancy [3]. Furthermore, mutations in anticoagulant or procoagulant protein associated genes play important roles in pathologic development of thrombophilia [4]. Genetic testing is useful for confirming diagnosis of hereditary thrombophilia.

Antithrombin (AT) is an endogenous anticoagulant that acts as a major clotting inhibitor by inactivating thrombin and factor Xa, IXa [5]. Inherited deficiency of AT is an uncommon autosomal dominant disorder with a 5 to 17 per 1000 individuals prevalence in the general population [3]. Mutations in the AT gene *SERPINC1* may cause two types of AT deficiency according to the absence of a variant AT in plasma (Type I) or the detection in plasma of a variant with impaired or null activity (Type II) [6].

Here, we report a case of hereditary thrombophilia manifested by recurrent thrombosis involving the deep veins of the lower extremities, splanchnic veins, and cerebral veins. In the past 7 years, the patient suffered multiple thrombosis-related diseases, including acute mesenteric vascular obstruction, cerebral venous sinus thrombosis, deep venous thrombosis (DVT) and pulmonary embolism (PTE). Treatment with dabigatran effectively controlled thrombosis until the patient developed multiple cerebral thrombosis due to self-discontinuation. In this visit, a novel heterozygous mutation (c.233_236dup, p.Val80Alafs*26) in *SERPINC1* was identified 7. Long-term dabigatran treatment was given, and no recurrence or side effects were found within the followed 5 years. All the information were collected from the patient after informing consent.

## Case presentation

**Fig. 1:**
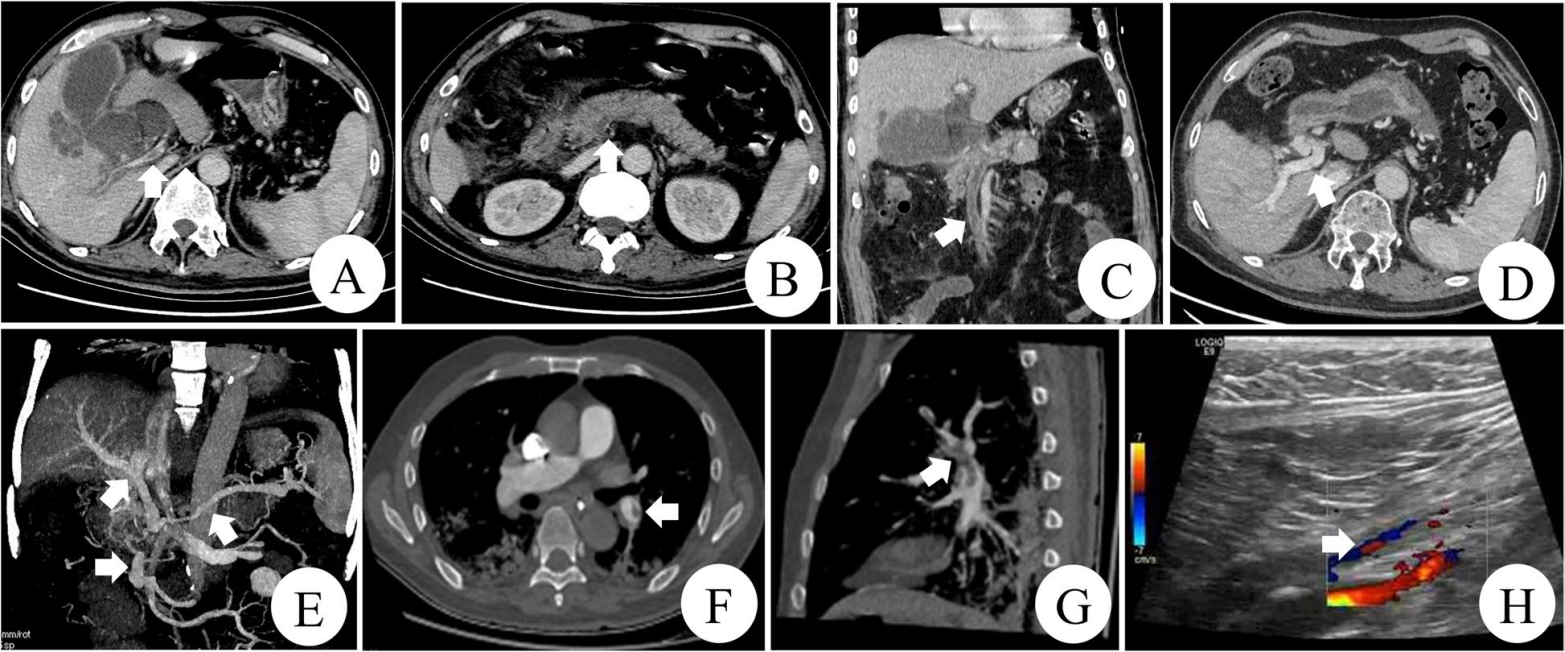
A triphase abdomen computed tomographic revealed filling defect in the major portal vein (**A**) and a low-density area in splenic vein (**B**). Coronal reconstruction of the abdominal computed tomography exhibited a low-density area in superior mesenteric vein (**C**). After 2 years of anticoagulation treatment with oral dabigatran, a triphase abdomen computed tomographic demonstrated complete recanalization of the portal vein thrombosis (**D**) and coronal maximum intensity projection showed the formation of aberrant collateral vessels and partial recanalization of the splenic vein, superior mesenteric vein thrombosis (**E**). At admission, transverse (**F**) and sagittal (**G**) computed tomography pulmonary angiography demonstrated filling defect in the left pulmonary artery. Color Doppler ultrasound showed thrombosis in bilateral femoral vein and the left popliteal vein

**Fig. 2:**
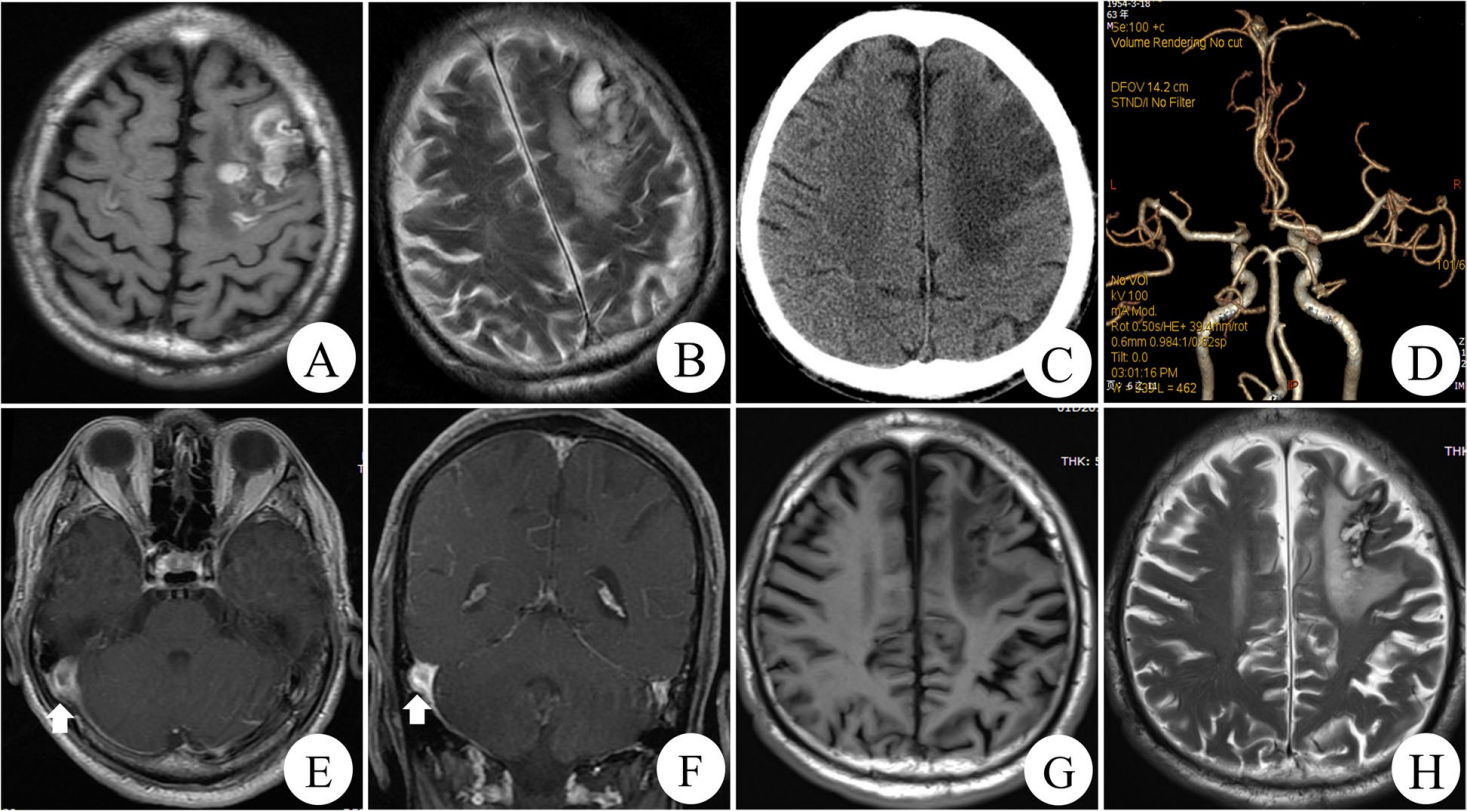
The T1-weighted magnetic resonance images (**A**), the T2-weighted magnetic resonance images (**B**) and CT images (**C**) demonstrate bilateral frontal hemorrhagic infarction. Axial (**E**) and coronal (**F**) contrast-enhanced T1-weighted magnetic resonance imaging demonstrate filling defect and a δ sign in the right transverse sinus, sigmoid sinus. The computed tomography angiography in brain is unremarkable except for hypoplasia of the left anterior cerebral artery A1 segment (**D**). The repeated T1-weighted magnetic resonance images (**G**) and the T2-weighted magnetic resonance images (**H**) reveal encephalomalacia in right frontal lobe after 2 years

**Fig. 3:**
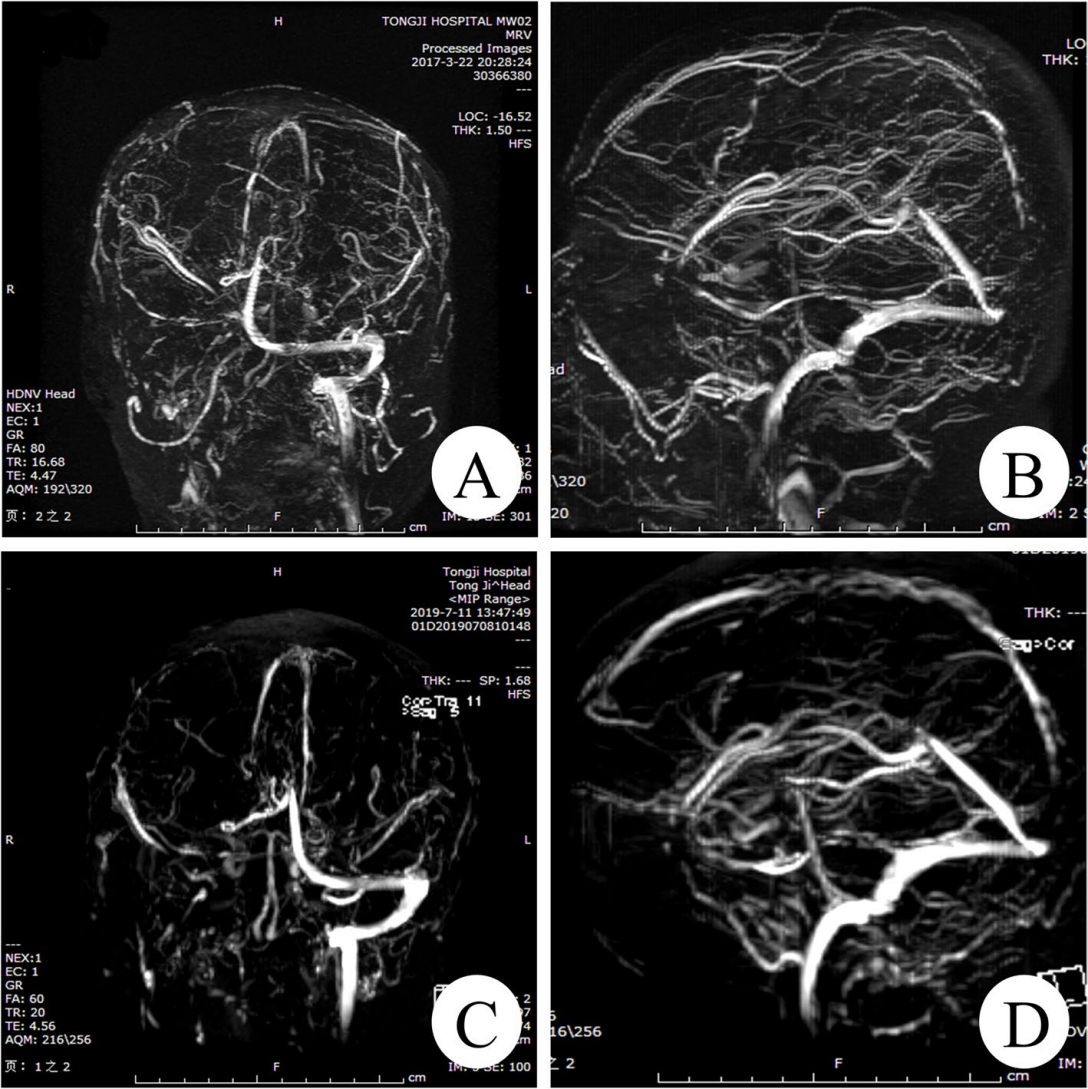
The brain magnetic resonance venography in 2017 reveals thrombosis involving right transverse sinus, right sigmoid sinus, superior sagittal sinus (**A**, **B**). The repeated magnetic resonance venography in 2019 demonstrates partial recanalization of right transverse sinus, right sigmoid sinus, superior sagittal sinus and the formation of aberrant collateral vessels (**C**, **D**)

A 61-year-old male patient was transferred to the gastrointestinal surgery department of our hospital for further treatment due to severe abdominal infection after he was performed a massive resection of small intestine for vascular ileus in December 2015. The results of the coagulation function test showed that the D-dimer (2.28ug/ml; reference range, 0-0.5ug/ml) was prolonged while thrombin time (TT), prothrombin time (PT) and active partial thromboplastin time (APTT) were normal. The plasma levels of AT antigen (58 mg/dl; reference range, 80-120 mg/dl) and AT activity (54%; reference range, 80–120%) were reduced. Abdomen enhanced computed tomography (CT) scan and computed tomography venography (CTV) revealed the several peripheral thromboembolus have been filled in the portal vein (Fig. 1 A), splenic vein (Fig. 1B), superior mesenteric vein (Fig. 1 C). Pulmonary artery CT angiography (CTA) demonstrated the massive thrombosis of the left pulmonary artery (Fig. 1 F, G). Color Doppler ultrasound showed thrombosis in bilateral femoral vein and the left popliteal vein, which suggested DVT in both lower extremities (Fig. 1 H). His family history was unremarkable except for sudden death of his nephew and niece from possible pulmonary embolism. He received standard thrombolytic therapy. Initially, he had achieved significant improvement in recanalization of the venous thrombosis. The patient was prescribed with oral dabigatran and was discharged. However, the patient was referred to the department of neurology for status epilepticus after ceasing dabigatran by himself in March 2017. Brain CT and magnetic resonance imaging (MRI) revealed bilateral frontal hemorrhagic infarction (Fig. 2 A, B, C). Brain enhanced MRI (Fig. 2E, F) and MRI Venography (MRV) (Fig. 3 A, B) documented multiple thrombosis in the right transverse sinus, sigmoid sinus, and superior sagittal sinus. CTA in head was unremarkable with no vascular malformation, aneurysm or stenosis (Fig. 2D). A total of 4 mg clonazepam was immediately administered intravenously as antiepileptic therapy. He received lamotrigine titrated to 75 mg bid and levetiracetam titrated to 1000 mg bid, after which clinical signs of seizure activity ceased. Simultaneously, he received standard anticoagulation treatment with low-molecular-weight heparin (LMWH) for the first 14 days once again, followed by the addition of oral dabigatran 110 mg Bid for 3 days, and then oral dabigatran only.

To identify the etiology of this patient showed multisystem VTE, serial blood tests were performed. Routine biochemical, renal, hepatic, blood lipid profile, homocysteine, thyroid profiles, tumor markers, electrocardiogram and transthoracic echocardiography were normal. The coagulation profile of the patient was determined, which included thrombin time, international normalized ratio (INR), fibrinogen degradation products, factor V, VII, VIII, IX, protein C, S, rheumatoid factor, lupus anticoagulant, antiphospholipid antibodies, anticardiolipin antibodies, antinuclear antibodies, and antineutrophil cytoplasmic antibodies, but no abnormality was found. The D-dimer (2.35 ug/ml; reference range, 0-0.5 ug/ml) was slightly elevated. The plasma levels of AT antigen (43 mg/dl; reference range, 80–120 mg/dl) and AT activity (33%; reference range, 80–120%) were found to be decreased.

**Fig. 4:**
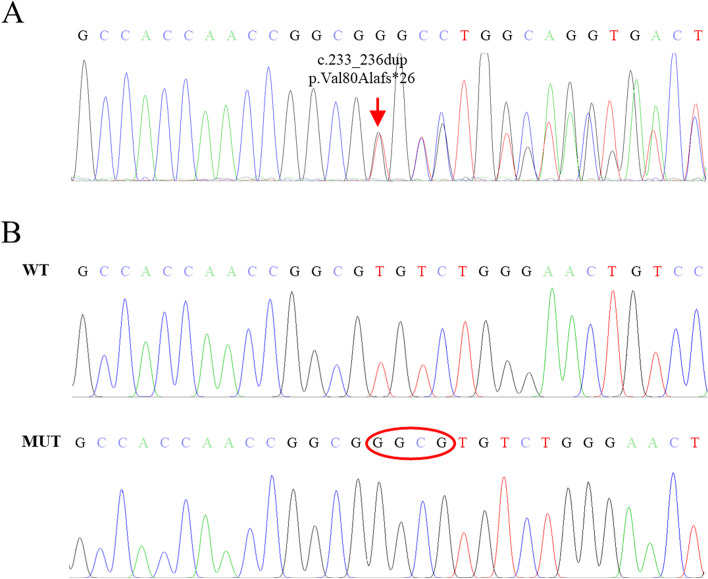
The heterozygous mutation in the *SERPINC1* gene (c.233_236dup, p.Val80Alafs*26) was identified in the patient. The red arrow indicates the start of the frameshift duplication in the *SERPINC1* gene (**A**). Sanger sequencing results of the TA cloned products (**B**). TA cloning and sanger sequencing were performed by Tsingke Biotechnology Co., Ltd. Chromas was used to analyze the sequencing results

**Fig. 5:**
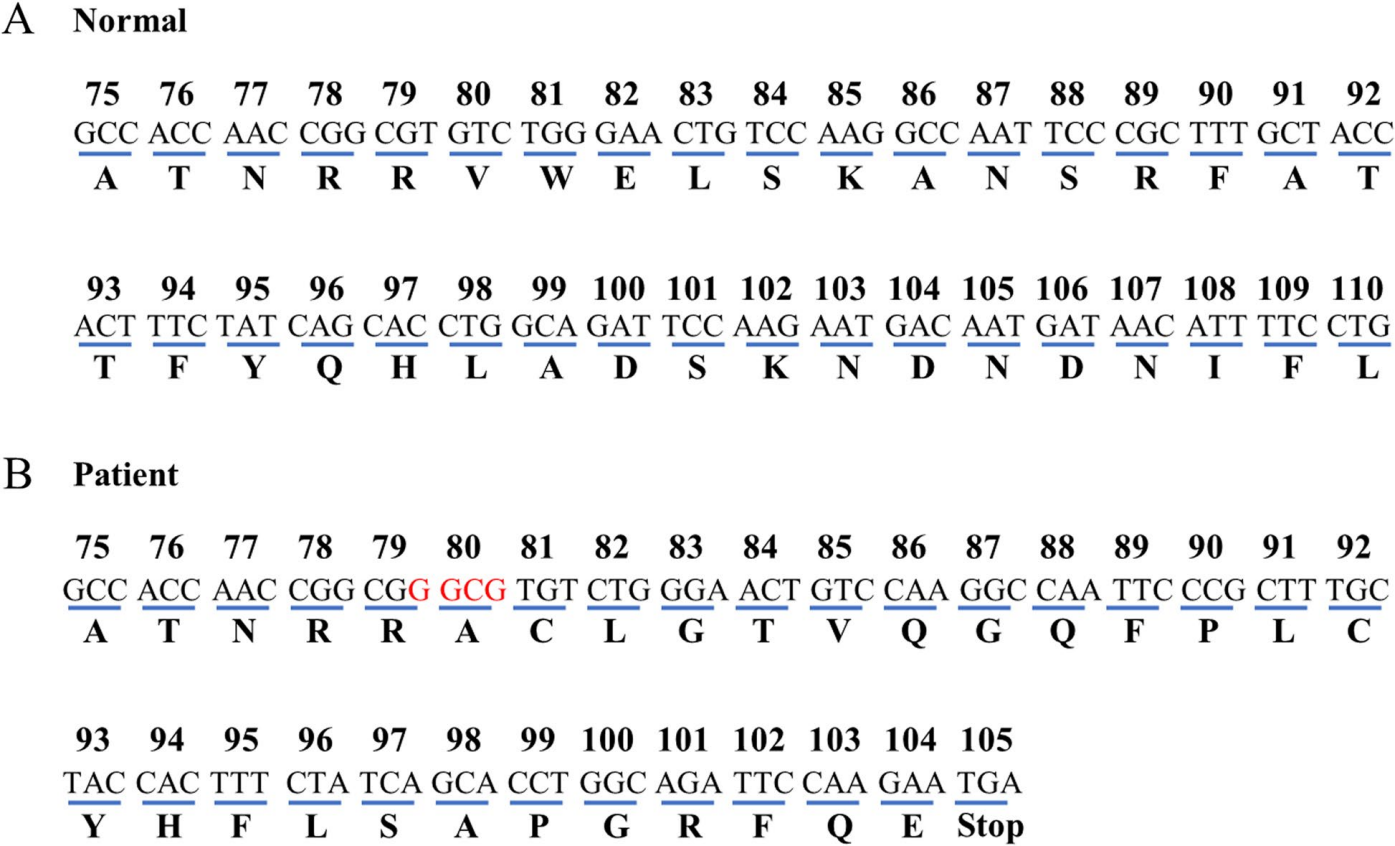
Part of the sequence (codons 75 to 110) for exon 2 of *SERPINC1* gene and the corresponding translated amino acids. Normal sequence showing the wild-type nucleotide sequence and the corresponding translated amino acids. *SERPINC1* coding region consists of 464 amino acids (**A**). Patient sequence showing the repeated nucleotides GGCG in codon 80 and the resulted premature stop-codon at codon 105 (**B**)

The patient was subjected to whole exome sequencing (WES) to identify potential pathogenic variants. A novel heterozygous frameshift duplication (c.233_236dup, p.Val80Alafs*26) in the *SERPINC1* gene was identified (Fig. 4A). To further confirm the mutation, the sample DNA was amplified by PCR (*SERPINC1* primers: 5’-TCTGCTTTACTGGGGCAACC-3’ and 5’-GTGCTCCTAACAAGGTGGCT-3’) and the PCR products were combined with the pClone007 Versatile Simple Vector (TSV-007VS, Tsingke Biotechnology Co., Ltd) by TA cloning. Subsequently, the isolated allele vectors were subjected to Sanger sequencing (Fig. 4B). The duplication mutation in *SERPINC1* caused a frame-shift mutation which resulted in a stop-codon at amino acid 105, leading to a premature termination of AT protein (Fig. 5).

The recovery of myodynamia and daily ability were significantly achieved within one year after the treatment. His seizures were kept under control by a combination therapy with lamotrigine and levetiracetam. Due to the inherited AT deficiency, oral dabigatran was continued with semiannual follow-up, including blood tests, Doppler ultrasound in the peripheral vessels and brain MRV. Blood tests revealed that the repeated measurement of plasma AT antigen and activity were constantly lower than normal. D-dimer was 0.22 ug/ml and other coagulation tests were unremarkable. The Color Doppler ultrasound showed bilateral superficial femoral veins, popliteal veins and their tributaries were partially recanalized after chronic thrombosis. Abdomen CTV in 2019 revealed recanalization of the portal vein, splenic vein, superior mesenteric vein thrombosis (Fig. 2D, E) At the same time, D-dimer and other coagulation tests were within the normal range, except for the reduction of plasma AT antigen and activity. The Color Doppler ultrasound in 2021 showed partial recanalization of bilateral superficial femoral vein and the popliteal vein thrombosis. Brain MRV improved even if recanalization was not completed after 2 years of the onset of the cerebral infraction (Fig. 3 C, D). The blood test results were not much different from the previous ones. The last follow-up visit in February 2022 indicated that the patient recovered significantly.

## Discussion and conclusions

Thrombophilia is a group of disorders in which blood has an increased tendency to clot. [3] The levels of AT antigen and AT activity were significantly reduced after the acute thrombosis had recovered, which demonstrated the reported patient had AT deficiency. Moreover, a mutation (c.233_236dup p.Val80Alafs*26) in the *SERPINC1* gene was identified by WES. The duplication mutation can lead to a frameshift at the 80th codon (Val) and premature termination at the 26th downstream amino acid of the *SERPINC1* protein (p.Val80Alafs*26), which will produce a truncated protein with 104 amino acids by prediction, also the premature stop codon will lead to mRNA decay (Fig. 5). As the DNA sequence of splice sites (GT and AG) are highly conserved, the frameshift duplication mutation inserts GGCG into DNA sequence, do not contain GT or AG bases, which will not affected the splicing of mRNA. Thus, we believe that multisystem VTE in this case should be associated with AT deficiency caused by *SERPINC1* mutation.

Up to 80% of cases with suspicion of inherited AT deficiency are caused by defects of *SERPINC1 *[8][9]. According to the Human Gene Mutation Database (HGMD), more than 250 mutations in the *SERPINC1* have been already identified, and missense or nonsense mutations constitute more than 50% to the genetic defects, followed by small deletions, gross deletions, and small insertions [10]. Type I deficiency is caused by nonsense mutations or short insertions and deletions within *SERPINC1* which lead to frameshifts and result in a failure in expression of AT (quantitative deficiency). Type II deficiency is usually caused by missense mutations affecting residues that are involved in AT function (qualitative deficiency) [11]. Clinically, the thrombotic events often occur at an earlier age if someone has hereditary AT deficiency. VTE occurred in 85% of AT deficient relatives before 55 years of age in family studies [12]. Moreover, homozygous individuals with type I AT deficiency have a higher risk of severe venous thromboembolism (VTE) in childhood [13]. However, this patient as well as part patients in other cases are older than 60 [14], suggesting that although AT deficiency is the strongest congenital thrombophilia, there is a considerable clinical heterogeneity in the mutation causing the deficiency, as well as the participation of additional risk or protective factors [15].

The initial management of VTE in patients with AT deficiency should generally be no different from its management in those without AT [16]. Occasionally, it is worthwhile to consider AT concentrate in the individual with severe thrombosis [17]. It is suggested that individuals carried with hereditary AT deficiency and already developed to VTE should receive long-term anticoagulation[16]. Based on this recommendation, a long-term oral anticoagulant dabigatran was applied in this case and no thromboembolic and bleeding event occur during administration of dabigatran. Dabigatran is a reversible, potent, competitive direct thrombin inhibitor. In comparison to similar anticoagulant strategy like warfarin, the benefits of dabigatran include decreased risk in major ischemic and bleeding event, the low extent of dietary and drug interactions as well as good compliance since there is no need of regularly laboratory test in monitoring clotting indices [18, 19]. More importantly, we have shown the successful treatment with dabigatran of multisystem VTE associated with hereditary type I AT deficiency. However, long-term effectiveness and safety of dabigatran for hereditary AT deficiency need to be confirmed in a big number of cases.

There are also some limitations in this case. A disadvantage of direct sequence analysis is that it is inadequate for revealing large gene rearrangements in all coding regions of *SERPINC1* gene. Multiplex Ligation-dependent Probe Amplification (MLPA) should be performed simultaneously. Genetic counseling for his whole family is necessary. We are not able to determine AT deficiency in the asymptomatic family members based on these genetic data.

In conclusion, this multisystem VTE patient presents a novel frameshift variation within the *SERPINC1* gene, which leads to a failure in expression of AT (type I AT deficiency), and oral anticoagulant dabigatran may be promising in prevention of VTE.

## Data Availability

All data generated or analyzed during this study are included in this published article.

## Abbreviations

APTT: active partial thromboplastin time
AT: antithrombin
CT: computed tomography
CTA: computed tomography angiography
CTV: computed tomography venography
DVT: deep venous thrombosis
INR: international normalized ratio
LMWH: low-molecular-weight heparin
MLPA: Multiplex Ligation-dependent Probe Amplification
MRI: magnetic resonance imaging
MRV: : magnetic resonance imaging venography
PT: prothrombin time
PTE: pulmonary embolism
TT: thrombin time
VTE: venous thromboembolism
WES: whole exome sequencing

## References

[1] Stevens SM (2016). Guidance for the evaluation and treatment of hereditary and acquired thrombophilia. J Thromb Thrombolysis.

[2] Dautaj A (2019). Hereditary thrombophilia Acta Biomed.

[3] Martinelli I, De Stefano V, Mannucci PM (2014). Inherited risk factors for venous thromboembolism. Nat Rev Cardiol.

[4] Connors JM (2017). Thrombophilia Testing and venous thrombosis. N Engl J Med.

[5] Patnaik MM, Moll S (2008). Inherited antithrombin deficiency: a review. Haemophilia.

[6] Teng XY (2020). A novel mutation of SERPINC1 in a patient presenting as recurrent cerebral sinus venous and portal vein thrombosis. Blood Coagul Fibrinolysis.

[7] Grundy CB (1991). Recurrent deletion in the human antithrombin III gene. Blood.

[8] Luxembourg B (2011). Molecular basis of antithrombin deficiency. Thromb Haemost.

[9] Olds RJ (1993). Complete nucleotide sequence of the antithrombin gene: evidence for homologous recombination causing thrombophilia. Biochemistry.

[10] Xiong HY (2015). RNA splicing. The human splicing code reveals new insights into the genetic determinants of disease. Science.

[11] Cooper PC (2011). The phenotypic and genetic assessment of antithrombin deficiency. Int J Lab Hematol.

[12] Narlı Özdemir Z, Özcan M (2017). Proper diagnosis of antithrombin III deficiency. Anatol J Cardiol.

[13] Provazníková D (2020). Seventeen novel SERPINC1 variants causing hereditary antithrombin deficiency in a czech population. Thromb Res.

[14] Nowak W (2022). New SERPINC1 gene mutations in patients with antithrombin deficiency: antithrombin Lodz I, II, III, and IV. Pol Arch Intern Med.

[15] Bravo-Pérez C (2020). Genotype-phenotype gradient of SERPINC1 variants in a single family reveals a severe compound antithrombin deficiency in a dead embryo. Br J Haematol.

[16] Haemostasis and Thrombosis Task Force and British Committee for Standards in Haematology (2001). Investigation and management of heritable thrombophilia. Br J Haematol.

[17] Bauer KA (2003). Management of thrombophilia. J Thromb Haemost.

[18] Eikelboom JW (2013). Balancing the benefits and risks of 2 doses of dabigatran compared with warfarin in atrial fibrillation. J Am Coll Cardiol.

[19] Redondo S (2011). Pharmacological basis and clinical evidence of dabigatran therapy. J Hematol Oncol.

